# Extensive Soot Compaction by Cloud Processing from Laboratory and Field Observations

**DOI:** 10.1038/s41598-019-48143-y

**Published:** 2019-08-14

**Authors:** Janarjan Bhandari, Swarup China, Kamal Kant Chandrakar, Greg Kinney, Will Cantrell, Raymond A. Shaw, Lynn R. Mazzoleni, Giulia Girotto, Noopur Sharma, Kyle Gorkowski, Stefania Gilardoni, Stefano Decesari, Maria Cristina Facchini, Nicola Zanca, Giulia Pavese, Francesco Esposito, Manvendra K. Dubey, Allison C. Aiken, Rajan K. Chakrabarty, Hans Moosmüller, Timothy B. Onasch, Rahul A. Zaveri, Barbara V. Scarnato, Paulo Fialho, Claudio Mazzoleni

**Affiliations:** 1 Atmospheric Sciences Program and Department of Physics, Michigan Technological University, Houghton, MI USA; 2 Atmospheric Sciences Program and Department of Chemistry, Michigan Technological University, Houghton, MI USA; 3 Pacific Northwest National Laboratory, Richland, WA USA; 4 Atmospheric and Oceanic Sciences, McGill University, Montreal, Canada; 5 Institute of Atmospheric Sciences and Climate (CNR-ISAC), Rome, Italy; 6 Institute of Methodologies for Environmental Analysis (CNR-IMAA), Rome, Italy; 7 School of Engineering - University of Basilicata, Potenza, Italy; 8 Earth & Environmental Sciences Division, Los Alamos National Laboratory, Los Alamos, NM USA; 9 Department of Energy, Environmental and Chemical Engineering, Washington University in St. Louis, St. Louis, MO USA; 10 Desert Research Institute, Reno, NV USA; 11 Aerodyne Research Inc., Billerica, MA USA; 12 DNV GL, Høvik, Norway; 13 Instituto de Investigação em Vulcanologia e Avaliação de Riscos – IVAR, University of Azores, Azores, Portugal; 14 Department of Chemistry and Institute for Atmospheric and Earth System Research (INAR), University of Helsinki, Helsinki, Finland

**Keywords:** Atmospheric science, Climate change

## Abstract

Soot particles form during combustion of carbonaceous materials and impact climate and air quality. When freshly emitted, they are typically fractal-like aggregates. After atmospheric aging, they can act as cloud condensation nuclei, and water condensation or evaporation restructure them to more compact aggregates, affecting their optical, aerodynamic, and surface properties. Here we survey the morphology of ambient soot particles from various locations and different environmental and aging conditions. We used electron microscopy and show extensive soot compaction after cloud processing. We further performed laboratory experiments to simulate atmospheric cloud processing under controlled conditions. We find that soot particles sampled after evaporating the cloud droplets, are significantly more compact than freshly emitted and interstitial soot, confirming that cloud processing, not just exposure to high humidity, compacts soot. Our findings have implications for how the radiative, surface, and aerodynamic properties, and the fate of soot particles are represented in numerical models.

## Introduction

Soot particles, optically defined as black carbon^1^, are ubiquitous in the atmosphere^2,3^. They are emitted during incomplete combustion of carbonaceous materials including fossil fuels and biomass^4^. Soot particles contain toxic material on their surface, and are considered carcinogenic^5^. Soot also strongly absorb solar radiation influencing the Earth’s radiative balance through aerosol-radiation interactions, aerosol-cloud interactions, and by changing the surface albedo and the atmospheric stability^6–8^. Soot represents one of the strongest positive anthropogenic radiative forcers, possibly second only to CO_2_^3,9,10^. The properties of soot particles depend on their evolution in the atmosphere; in particular, freshly emitted soot particles are fractal-like lacy aggregates, composed of nanometer-sized monomers^11–16^; however, aged soot particles often have more compact morphologies. This transformation affects the particles’ optical, aerodynamic, and surface properties^16–21^. Therefore, understanding these transformations is key to accurately represent the dynamic properties of soot in climate and air-quality models.

Freshly emitted soot is typically hydrophobic^22–26^ but becomes hydrophilic over time due to condensation of organic or inorganic compounds, and coagulation with other particles^15,16,19,27–30^. Atmospheric oxidizing agents such as ozone, hydroxyl radicals, and nitrogen oxides promote the formation of oxygen-containing polar functional groups (e.g., carboxylates) on the soot surface, also making it more hydrophilic^31–34^. A detailed discussion on the water uptake by soot aggregates, based on surface polarity, from different fuel sources can be found in Popovicheva *et al*.^35^. Hydrophilic soot particles can act as cloud condensation nuclei (CCN) at atmospherically relevant supersaturations^24,36^.

Coating material on the surface of soot, including water, exerts capillary forces between the monomers and can cause the aggregate to restructure to a more compact morphology^19,20,37^. Some researchers proposed that soot compaction occurs during the condensation of water^38–40^, while others argued that the compaction occurs during evaporation^24,41,42^. China *et al*.^18^ found that a significant fraction of soot collected in the North Atlantic free troposphere was very compact and hypothesized that the compaction was due to cloud processing during long-range transport in the atmosphere. In an experiment with diesel soot, Huang *et al*.^43^ conducted up to three cycles of water condensation-evaporation on soot particles and observed restructuring. They also suggested that their findings represent only a lower limit for soot compaction during cloud processing and hypothesized that their observations of compact soot aggregates in the Grand Canyon, USA was due to cloud processing. Analyzing ambient samples collected during smoke periods (ship and biomass burning emissions), Shingler *et al*.^37^ reported compaction of soot particles upon humidification. They found that compaction was higher at 95% compared to 85% RH, implying additional shrinkage at higher relative humidity. Similarly, Lewis *et al*.^44^ found that soot particles in ambient smoke samples (at around 20% RH) underwent remarkable compaction after humidification (~80% RH). Earlier, in experiments conducted on human beings, Chamberlain *et al*.^45^ found that exhaled soot particles were compact upon humidification in the respiratory tract compared to the inhaled lacy aggregates. A more recent cold cloud processing laboratory experiment also showed that lacy soot aggregates become compact after super-cooled water condensation, and even more after ice nucleation^17^. The authors also found that compaction affects the soot optical properties. In fact, light absorption and scattering change when a soot particle undergoes morphological transformations, ultimately affecting the soot radiative forcing^17–19,21,44,46,47^. These lines of evidence suggest that water condensation or evaporation on soot particles changes extensively their morphological, porosity and surface properties, with implications for their effects on climate and human health. However, a quantification of these morphological changes for different atmospheric conditions using simple parameters that can be used in numerical models is still lacking.

In our study, we survey the morphology of several thousand soot particles using electron microscopy. We use a few basic morphological parameters and draw some general conclusions. Because the main goal of this paper is to discuss the morphological changes induced during cloud processing, we first focus our attention on samples collected at a site in the Po Valley of Italy, where fog and soot particles are abundant. To further quantify the process, we simulated some of the ambient conditions in the Michigan Tech Pi Cloud Chamber laboratory facility. We discuss the laboratory results to understand the role of water activation and humidification, and to explore bounds for the soot compaction. Finally, we summarize the morphological properties of soot collected at eleven locations around the world characterized by different sources, time since emission, and atmospheric processes.

## Results

### Morphology of San Pietro Capofiume soot particles

**Figure 1:**
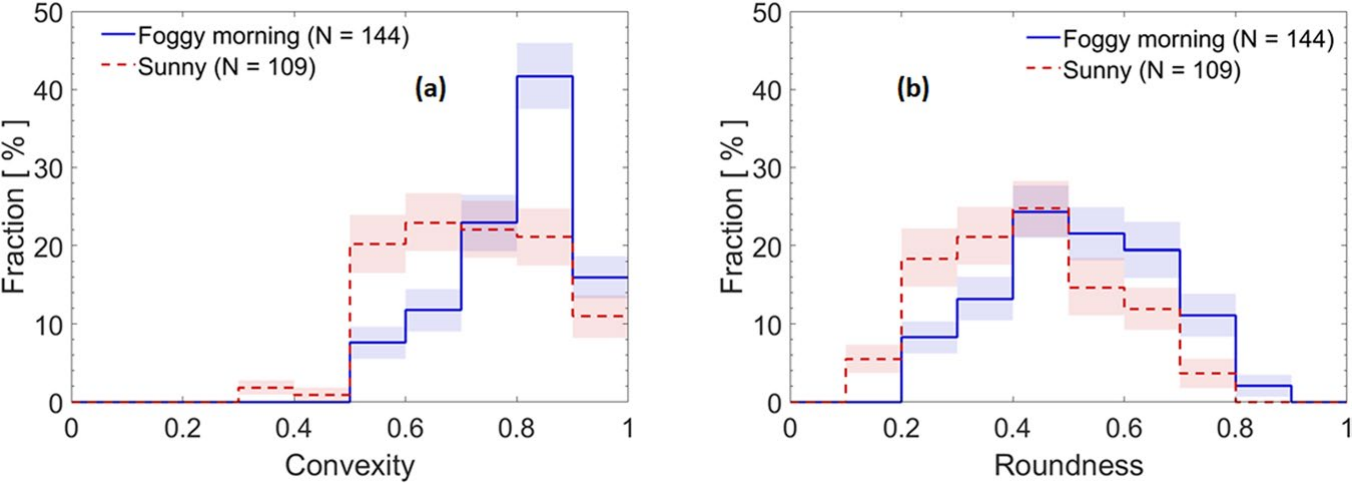
Convexity and roundness of soot particles from the San Pietro Capofiume site in the Po Valley, Italy. Distributions of (**a**) convexity and (**b**) roundness for soot particles of categories C0 and C1. The colored bands represent 68% confidence intervals (see the method section). The total number fraction of particles for each distribution is normalized to 100%.

From San Pietro Capofiume (SPC), a site in northern Italy’s Po Valley, we selected two samples: (1) a sample collected during a dense foggy morning event, and (2) a sample collected during a sunny event (see method section). During the image analysis of soot, we noticed that many particles were compact with and without visible coating material. Because we quantify compaction with morphological parameters (convexity, roundness, aspect ratio, and area equivalent diameter are defined in the method section) and because residual coating material, such as organics, can affect the parameters estimated from the 2D projected images, we focused only on soot particles with a small amount of coating (categories C0 – bare or thinly coated, and C1 – partly coated, as discussed in the SI). This choice maximizes the chances of quantifying cloud-induced compaction by excluding particles compacted by coating material different from water (with the exception for soot particles coated by other material after the water evaporated). Most of the imaged soot particles were coated (Supplementary Fig. S4). Aerosol mass spectrometry data showed a large mass fraction (29–46%) of organics, along with nitrate (17–42%), ammonium (7–15%), and sulfate (4–7%) in non-refractory PM_1_ that might have contributed to the coating on the soot particles and to the soot hydrophilicity (Supplementary Figs. S6 and S7). The fraction of C0 and C1 soot particles was higher during the sunny than the foggy morning event (48% vs. 21%). Only a minor fraction of soot particles (<6%) were partially encapsulated by, or attached to, other materials. We compared the C0 and C1 soot from the sunny event to that from the foggy morning, to find evidence of cloud processing in their morphologies. A total of 109 individual soot particles were imaged and analysed for the sunny event, and 144 soot particles were imaged and analysed for the foggy morning event. In Fig. 1a,b we show convexity and roundness distribution plots, respectively (both parameters increase with increasing compaction). It is evident that both distributions are shifted toward larger values for the foggy morning sample with respect to the sunny sample.

Consistently with these findings, the aspect ratio and area equivalent diameter distributions of the soot particles shifted to lower values (Supplementary Figs. S9 and S10), while the soot monomers did not show significant size changes (Supplementary Table S2), suggesting similar emission sources and consistent with the low amount of coating in the C0 and C1 categories. The means, standard deviations, standard errors, and total errors for different morphological parameters are summarized in Supplementary Table S2. These findings support the hypothesis that ambient soot compaction can indeed arise from cloud processing alone.

### Morphology of soot particles from the Pi Chamber

**Figure 2:**
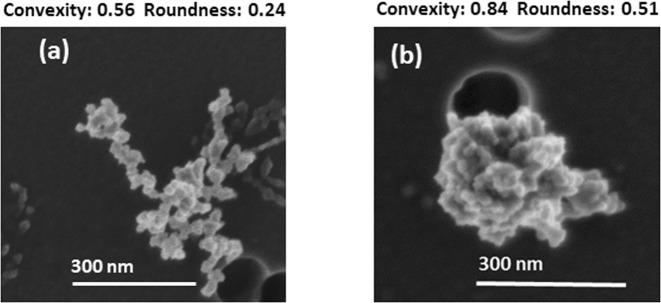
Scanning electron microscopy micrographs of interstitial and residual soot particles collected from the Pi Chamber. Soot particles were collected on polycarbonate membranes and imaged at an accelerating voltage of 1 kV, an emission current of 10 μA, and a working distance of 4 mm: (**a**) interstitial soot particle of convexity 0.56 and roundness 0.24 (magnification of 90 kX), and (**b**) residual soot particle of convexity 0.84 and roundness 0.51 (magnification of 100 kX). The dark spots are pores in the membranes.

To study the soot compaction process under controlled conditions, we performed experiments in the laboratory. We utilized a turbulent cloud chamber, referred to hereafter as the “Pi Chamber” (briefly described in the method section) to subject soot particles to cloud processing. We collected three types of soot particles: 1) particles on which water nucleated into droplets but had been dried before sampling (residual), 2) particles that had been subjected to high RH conditions but were not inside a water droplet at the time of collection (interstitial), and 3) particles just emitted by the combustion source and not yet sent to the Pi Chamber (nascent). Residual soot particles from cloud droplets showed clear morphological compaction with respect to interstitial and nascent soot particles (Fig. 2). We note that both the interstitial and residual particles were exposed to RH values near 100%.

**Figure 3:**
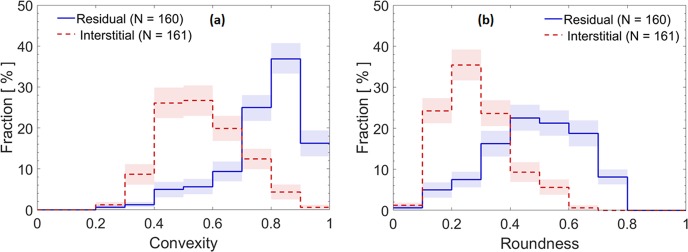
Convexity and roundness of soot particles from the Pi Chamber. Distribution of (**a**) convexity, and (**b**) roundness for residual and interstitial soot particles. The colored bands represent 68% confidence intervals. The total number fraction of particles for each distribution is normalized to 100%.

Convexity and roundness were significantly higher for the residual compared to the interstitial samples, indicating substantial compaction of soot particles by cloud processing, as shown in the distribution plots in Fig. 3.

These findings are substantiated by a decrease in aspect ratio and area equivalent diameter for the cloud droplet residuals, as clearly visible in the distribution plots shown in the SI (Supplementary Figs. S13 and S14). The large range in area equivalent diameter reflects the polydisperse size distribution of the soot particles generated during the experiments that were injected into the chamber without size selection. However, no significant change in the size of monomers was detected.

We also compared the morphology of soot particles for nascent and interstitial samples. Their probability distributions mostly overlap (Supplementary Figs. S15 and S16) suggesting that the high RH alone did not result in soot compaction; in other words, only those particles that activated to cloud droplets (residuals) compacted. This result contrasts with some previous studies that found soot to compact at high, but sub-saturated, RH conditions^19,37,44,48^. The discrepancies between our and previous studies might be due to different degrees of soot aging.

**Figure 4:**
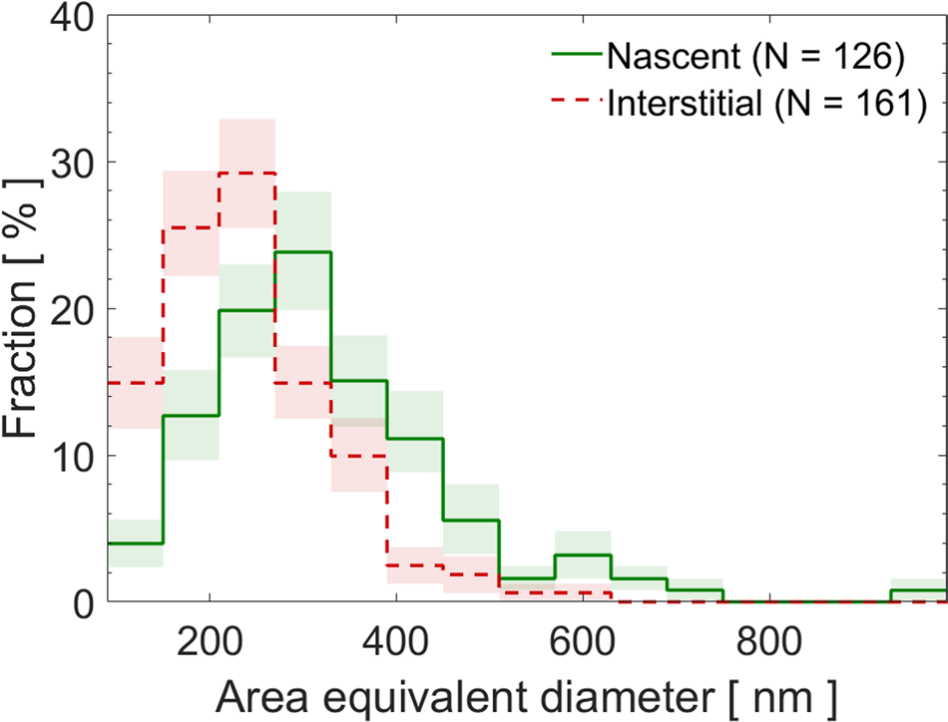
Distribution of the area equivalent diameter of nascent and interstitial soot particles from the Pi Chamber. The colored bands represent 68% confidence intervals. The total number fraction of particles for each distribution is normalized to 100%.

An unrelated but interesting result is that the distribution of the area equivalent diameter for the interstitial soot is shifted toward smaller sizes compared to that of nascent particles, indicating that water droplets nucleated preferentially onto larger particles^49^ (Fig. 4).

The average monomer diameters for the soot particles from the Pi Chamber were smaller than those of the ambient soot (Tables S2 and S4). The size of the monomers in soot aggregates depends on various factors like flaming conditions, fuel type etc.^50,51^, and atmospheric aging. The soot particles sampled in the Pi Chamber were fresh (collected within an hour from emission), while the particles sampled at the SPC site were mostly aged. However, we also note that the differences in the averaged monomer diameters in our samples are not statistically significant. Finally, the size distribution of nascent soot generated by kerosene combustion during the Pi Chamber experiments are remarkably similar to the size distribution of the soot particles collected during the sunny event at SPC (Figs. 4 and S10).

## Discussion

The Pi Chamber experiments confirmed that soot was compacted during cloud processing and not because of high RH conditions. Interestingly, the RH during the period of the “sunny” sample from the San Pietro Capofiume campaign remained above 75%, meaning that the particles were humidified and yet the soot morphology was markedly different with respect to the “foggy morning” case. However, the Pi Chamber samples showed much greater changes in morphology compared to the SPC ambient samples. This observation is consistent with the fact that the ambient samples are a complex mixture of soot of different degrees of aging and processing, compared to the chamber experiments. In addition, we had no means to separate interstitial from residuals during the SPC ambient sampling. During the sunny event, pre-existing soot might already have been cloud processed by a previous fog event. Therefore, it is reasonable that the roundness and the convexity of the ambient soot for the sunny event were higher than those of the interstitial particles collected from the Pi Chamber. It is interesting to note that the roundness and convexity distribution plots for the foggy morning event (blue shades in Fig. 1) and those for the cloud droplet residuals from the Pi Chamber (blue shades in Fig. 3) are quite similar. This suggests that the right side of these convexity and roundness distributions might represent upper limits for these parameters in warm cloud conditions and for short processing times. The mean values of convexity and roundness are also comparable to the mean values of convexity (0.75) and roundness (0.45) observed by China *et al*.^17^ in diesel soot residuals from liquid water droplets. The values presented here are, however, lower than the values obtained for ice crystal residuals (convexity = 0.83 and roundness = 0.55) reported by China *et al*.^17^, suggesting that ice nucleation might further compact soot. These observations might explain why the mean values of roundness and convexity for the SPC and Pi Chamber cloud processed soot are slightly lower than the convexity and roundness of soot samples retrieved at the Pico Mountain Observatory in the Azores, Portugal^18^. In their study, the particles were transported in the marine free troposphere at heights that might have resulted in freezing for at least some of the soot, resulting in more compact particles in the overall soot population. In addition, the soot analysed in their study was transported for several days in the atmosphere, allowing for multiple cycles of cloud processing. Therefore, both cloud processing type (cold vs. warm) and transport time probably play a role in determining the upper limit of soot compaction in the atmosphere.

**Figure 5:**
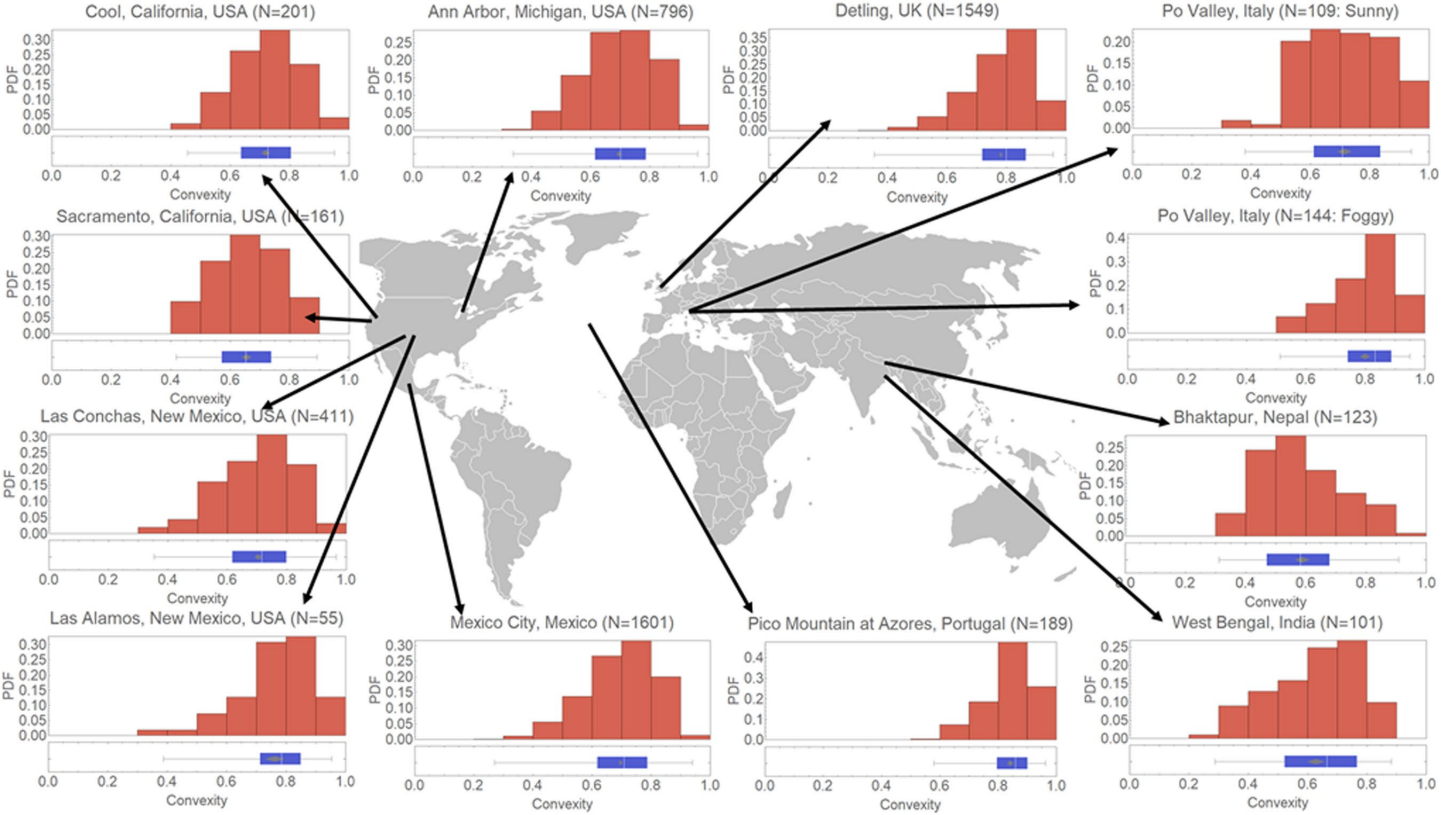
Convexity Probability Distribution Functions (PDFs) and box plots for C0 and C1 soot particles from different locations. In each box plot, the vertical white line represents the median and the grey diamond represents the mean confidence interval for each distribution, the box sides represent 25% and 75% quantiles and the whiskers represent the lower and upper extremes. For each distribution, N (in brackets) is the number of soot particles analysed.

To further put these results into a broader context, we investigated the morphology of ambient soot particles collected from different locations around the globe. We present the convexity results in Fig. 5 (a similar map for roundness is shown in Supplementary Fig. S17). Each histogram represents the probability distribution function of convexity. It is to be noted that the roundness and convexity plots were constructed only from soot with a small amount of coating (C0 and C1 categories), as for the data shown in the rest of the paper. In Table 1, we present the mean values of roundness and convexity for these ambient soot samples along with some laboratory results. The values are sorted by increasing convexity. Estimated sample age since emission and potential for participation in cloud processing are also reported. We should note that the time scale is only a semi-quantitative estimate.

**Table 1:** Mean values of roundness, convexity and area equivalent diameter (D_Aeq_) of ambient and laboratory soot particles (soot category C0 and C1).

Convexity	Roundness	D_Aeq_ [nm]	Sampling location, probable dominant source (sampling date, estimated sample age^*^)	Potential for cloud processing	N	Literature
0.56	0.29	239	Michigan Tech Pi Chamber, interstitial kerosene soot (January 2017, ~minutes)	low	161	This study
0.59	0.32	323	Bhaktapur, Nepal, brick kiln oven and road traffic (March 2017, ~minutes)	low	123	This study
0.63	0.36	125	West Bengal, India, urban (January 2018, ~minutes/mixed)	low	101	This study
0.65	0.38	324	Sacramento, California, urban (CARES, June 2010, ~minutes/mixed)	low	161	This study, Sharma *et al*.^69^ and Zaveri *et al*.^52^
0.70	0.40	222	Ann Arbor, Michigan, road traffic (July–August 2010, ~minutes/mixed)	low	796	China *et al.*^54^
0.70	0.41	410	Los Alamos, New Mexico, Las Conchas Fire plume (July 2011, <2 hours)	low	411	China *et al.*^57^
0.70	0.41	257	Mexico City, urban (MILAGRO, March 2006, ~minutes/mixed)	low	1601	This study and China S.^85^
0.71	0.41	153	Pacific Northwest National Laboratory, Washington nascent diesel soot (November 2013–January 2014, ~minutes)	low	226	China *et al.*^17^
0.71	0.43	326	Po Valley, Italy, sunny day, urban outflow and road traffic (December 2015, ~minutes/mixed)	low	109	This study
0.72	0.42	237	Cool, California, urban outflow and road traffic (CARES, June 2010, ~hours)	low	201	This study, Sharma *et al*.^69^ and Zaveri *et al*.^52^
0.75	0.45	179	Pacific Northwest National Laboratory, Washington, supercooled water droplet residuals from diesel soot (November 2013–January 2014, ~minutes)	high	208	China et al.^17^
0.76	0.45	330	Los Alamos, New Mexico, Whitewater-Baldy Complex Fire plume (May 2012, ~several hours)	low	55	This study and Girotto G.^86^
0.78	0.47	224	Detling, UK, London and Benelux outflows (January 31^st^, Benelux; February 2–3, London, 2012, ~several hours)	medium	1549	This study and Girotto G.^86^
0.78	0.48	192	Michigan Tech Pi Chamber turbulent cloud, residual kerosene soot (January 2017, ~minutes)	high	160	This study
0.80	0.52	237	Po Valley, Italy, foggy morning, urban outflow and road traffic (December 2015, ~minutes/mixed)	high	144	This study
0.83	0.55	201	Pacific Northwest National Laboratory, Washington, ice crystal residuals from diesel soot (November 2013–January 2014, ~minutes)	high	241	China *et al.*^17^
0.84	0.58	248	Pico Mountain Observatory, Azores, long range transport (July 2012, ~1 week)	high	189	China *et al.*^18^

^*^With the term “mixed” we indicate the potential presence of soot particles carried over from earlier emissions and mixing with fresher emissions. N is the number of single soot particles analyzed.

The data are sorted by increasing convexity.

It is evident that convexity and roundness of soot particles increase with aging time and the potential for cloud processing. Supplementary Figs. S18 and S19 summarize how the two morphological parameters increase with soot aging. For all time scales, the cloud processed soot particles have the highest convexity and roundness values, substantiating the primary role of cloud processing in soot compaction. Freshly emitted ambient soot particles collected in Bhaktapur, Nepal and West Bengal, India had among the lowest values of roundness (0.32 and 0.36) and convexity (0.59 and 0.63). Samples in Bhaktapur were collected near roads around brick kiln sites dominated by fresh emissions (less than a few minutes). Samples collected from a rural site in West Bengal, India, showed slightly higher values probably due to the slightly more aged particles (several minutes). Ambient soot particles collected during the 2010 Carbonaceous Aerosol and Radiative Effects Study (CARES)^52^ in the urban area of Sacramento, California, USA, showed slightly higher values; while samples collected during the same campaign but from the foothills of the Sierra Nevada Mountains in Cool, California, USA, showed even higher values, consistent with the longer aging times (several hours). The roundness and convexity of ambient soot from an urban site near downtown Mexico City (collected during the Megacity Initiative: Local and Global Research Observations (MILAGRO) campaign)^53^ were comparable to those from Sacramento, which is reasonable considering the similar aging times. Fresh samples (a few seconds from emission) collected at a road site in Ann Arbor, Michigan, USA^54^, also showed comparable values of roundness and convexity to the samples collected during the MILAGRO and CARES, but somewhat surprisingly higher than those from Bhaktapur, Nepal and West Bengal, India. The higher values might be due to the presence of soot emission from heavy-duty vehicles on the road. China *et al*.^54^ have found that the fractal dimension of soot increases with the fraction of heavy-duty vehicles. Additionally the convexity and roundness of lacy aggregates weakly decrease with the aggregate size^54^; the particles collected in Ann Arbor were among the smallest found for ambient samples (note the area equivalent diameter in Table 1). Finally, pre-existing soot particles might have been transported to the sampling sites from other surrounding sources. For ambient soot collected in Detling, UK, during the Clean air for London (ClearfLo) campaign^55^, the values were high consistent with somewhat long aging times for the air masses originating from the outskirts of London and the Benelux region. The higher values of roundness and convexity of the soot particles in Detling compared to those of Mexico City and California (Sacramento and Cool) might also be due to the moist weather conditions (winter) in the UK compared to the generally dry atmosphere of Mexico City and California. In a separate study, Wang *et al*.^15^ reported values of roundness (0.39) and convexity (0.70) for partly coated soot samples similar to our samples from Mexico City and California (Cool). They collected soot samples at a mountain site in a polluted area in the North China Plain during dry haze days in winter (low RH < 65%). Soot compaction was also observed for two fire events, the Whitewater-Baldy complex fire^56^, and the Las Conchas fire (samples were collected at the Los Alamos National Laboratory)^57^. Both roundness and convexity were higher for the more-aged soot from the Whitewater-Baldy fire. As mentioned earlier, the highest values of roundness and convexity were found for soot collected in the free troposphere at the Pico mountain observatory^18^ probably due to the long transport time (several days) from the source (typically in North America), and the likely multiple cloud processing cycles, potentially including ice formation.

We point out that several other factors can be important for the compaction process. For example, the soot surface chemistry (including thin coatings of different origins and aging) can impact the soot ability to interact with water, altering its wettability and ability to act as CCN. Additionally, the pH of the cloud droplets might also affect the compaction. With the data available for this study, we can only speculate on the effects of water pH on soot morphology. If there is an effect, it must be mediated by soot surface functional groups. These may comprise groups such as anhydrides, which can hydrolyze to carboxylic acids and partly dissociate when particles take up water becoming fog droplets, in which the pH would increase^58^. However, this process could also increase hydrogen bonding between the elements on the soot surface and stabilize the soot inclusions inside the droplets. These hypotheses should be the subject of future laboratory studies where the soot surface or the water pH could be varied while analyzing the effects on the soot compaction.

Our results have implications for how the properties of soot particles transported in the atmosphere should be represented in numerical models for climate and air quality applications. In fact, in recent ice nucleation studies, Mahrt *et al*.^59^, and Nichman *et al*.^60^ found that the ice nucleation ability of soot is affected by the availability of mesopores, suggesting that soot compaction might change its ice nucleation activity by affecting the number of mesopores. Additionally, several studies have shown that the optical properties of soot change when the soot becomes compact, with the effect of compaction being more pronounced for light scattering than for absorption^17,18,46,61^. Compact soot also has a higher effective density than lacy soot^62^, which can have an effect on dry deposition and electrical mobility. For example, the deposition of fibre-like particles in the lungs, such as for fresh soot, is enhanced compared to compact particles of the same mass, due to higher drag^63,64^. Finally, heterogeneous reactions on soot particles have shown to be affected by changes in their surface area because of compaction^65^. Related to this last issue, studies have shown that the toxicity of inhaled aerosol, including soot particles, increases with the surface area of the particles^66,67^, while in a recent study in Japan, Kiriya *et al*.^68^ showed that fresh soot concentrations correlated with aerosol surface area measurements, with the correlation weakening for aged soot.

## Methods

### Morphology and mixing state of soot

The focus of our study was to quantify the effects that water has on the soot morphology – not other coating material such as organics – therefore, we wanted to analyze the morphological descriptors only for soot particles that appeared to have little coating in the electron microscopy images. However, often, ambient soot particles are coated by different materials (other than water). Some soot particles are so thickly coated that the monomers are not clearly distinguishable in scanning electron microscopy images, this can bias the calculation of the morphological parameters, for example, if organic material fills the voids between monomers. Therefore, to achieve our goal, for ambient samples, we first analysed the mixing state of soot particles and classified them into four categories based on a visual inspection of the coating thickness: C0, C1, C2, and C3 as detailed in the SI and discussed elsewhere^57,69^. The results of such classification are provided in section 3 of the SI. From this classification we then selected only the soot belonging to categories C0 and C1, as mentioned in previous sections. Soot particles generated in the laboratory were freshly emitted with no visual coating and were all in the C0 category.

To quantify the structural changes that soot underwent during cloud processing and assess the degree of compaction, we investigated several morphological parameters. These include roundness, convexity, aspect ratio (*AR*), and area equivalent diameter (*D*_*Aeq*_). Detailed descriptions of these parameters and the limitations of image processing and analysis can be found elsewhere^54,57,70^ but we will briefly summarize the meanings of these parameters next. Roundness is the ratio of the projected area of an aggregate (*A*_*p*_) to the area of a circle having a diameter equal to *L*_*max*_, the maximum length of the aggregate, and is a measure of the particle geometry and topology. Convexity is the ratio of *A*_*p*_ to the area of the convex hull polygon inscribing the aggregate and is a topological property of the particle. *AR* is the ratio of *L*_*max*_ and the width of the projected aggregate (*W*) orthogonal to *L*_*max*_, and is a measure of the elongation of the particle. *D*_*Aeq*_ is the diameter of a spherical particle with a projected area equivalent to *A*_*p*_ and provides a quantitative measure of the particle size. The three parameters *AR*, convexity, and roundness incorporate different and somewhat complementary information. For example, a spherical particle has convexity, roundness, and *AR* values each equal to unity, while a rectangular parallelepiped laying on its long side has a convexity of unity, but its roundness is lower than one and its *AR* is larger than one. An example of the calculation of *AR*, convexity, and roundness of a soot particle is shown in Supplementary Fig. S1. Specifically, lacy soot particles with an open elongated structure are expected to show lower convexity and roundness, and higher *AR* values, with respect to compact soot particles. This is the case even if the particles (compact or lacy) have the same mass and identical monomer diameters. Additionally, when a soot particle becomes compact, we expect the *D*_*Aeq*_ to decrease.

To calculate the mean and confidence intervals for each bin in the distributions shown in Figs. 1, 3 and 4, as well as in the SI, we used a bootstrap method, in which frequency distributions are constructed from the raw data with 100,000 resampling with replacement^71^. The colored bands represent 68% confidence intervals for each bin.

Finally, we note that to quantify the morphology of soot, we also calculated the fractal dimension *(D*_*f*_) of soot particles. However, the *D*_*f*_ calculation methods, which are ensemble based, require a statistically significant number of soot particles that underwent similar processing^72^; therefore, a quantitative determination of *D*_*f*_ for ambient particles is uncertain and is not discussed here any further (*D*_*f*_ estimates are reported in the Supplementary Information).

### Ambient samples from San Pietro Capofiume (SPC)

We collected 13 ambient samples at a rural site in San Pietro Capofiume (SPC), in the Po Valley in Northern Italy, during a campaign period of one month in November-December of 2015. The low local temperature and high RH typical of the fall season, result in stable atmospheric conditions, favoring fog formation that interacts with anthropogenic pollutants present in high concentrations in the region^73,74^. We collected ambient particles onto 13 mm diameter polycarbonate filter membranes (pore size of 0.1 μm, Whatman Inc., Chicago, Illinois, USA) and 3 mm diameter lacy formvar copper grids (300 mesh copper, Ted Pella, Inc., Redding, California, USA) by using an aspiration technique that is described elsewhere^17,54^. During sampling, ambient air was drawn through a PM_2.5_ inlet using a diaphragm vacuum pump (Hargraves Technology Corporation, New Hampshire, USA). The flow rate varied between 0.12 and 0.26 lpm; however, it was nearly constant during each sampling period. Out of the 13 samples, we used five sample sets collected during different atmospheric conditions (Supplementary Figs. S2 and S3). Four sample sets were collected during fog conditions. Foggy events were characterized by low solar irradiance (<300 W m^−2^) and high Liquid Water Content (LWC > 0.08 g m^−3^). The LWC was measured with a Particulate Volume Monitor PVM-100^75^. As a reference, another sample set was collected during a sunny event (on November 30^th^) and was characterized by higher solar irradiance (~400 W m^−2^), close to the peak solar irradiance (~456 W m^−2^, see Supplementary Fig. S3) and LWC below the detection limit (<0.01 g m^−3^). Supplementary Table S1 provides details on sampling times and conditions. We used all five samples to study the soot mixing states. Out of these five samples, two were used to study the soot compaction described in this paper. We chose a sample during a dense foggy period in the morning (on December 4^th^) that we refer to as “foggy morning event”, with a fairly stable and elevated LWC ~0.11 g m^−3^. We compared the morphology of soot from this sample with the soot collected during the sunny event. We should mention, that the PM_2.5_ inlet did not allow us to collect soot particles that were solely residuals of fog droplets and therefore, a clear distinction between residuals and interstitials was not possible. However, soot particles from the foggy morning event were still expected to be more likely processed by the fog, while the soot particles from the sunny event were expected to contain a larger fraction of soot not yet processed. The two sample sets were collected approximately at the same time of the day. Sulfur/carbon atomic ratios for coated soot were quantified in those two samples using a computer controlled scanning electron microscope (CCSM) coupled with Energy-Dispersive X-ray spectroscopy (EDX) (Quanta 3D model, FEI, Inc.). The EDX spectra were acquired for 10 seconds of live time, at an accelerating voltage of 20 kV and a beam current of ~500 pA. Lacy-type transmission electron microscope (TEM) grids were used for EDX to reduce substrate carbon signals. A total of 204 and 426 internally mixed soot particles were analyzed with EDX for the sunny event and foggy morning event samples, respectively.

For single particle imaging and classification, we used the samples collected on polycarbonate membranes. The membranes were coated with 1.5 nm (±10%) thick layer of Au/Pd alloy in a sputter coater (Cressington 208HR) and we imaged individual particles with a Hitachi S-4700 field emission scanning electron microscope at a magnification of 60–100 kX, an accelerating voltage of 1 kV, and a working distance of 4 mm. We also captured images using an environmental transmission electron microscope (FEI, Inc. model Titan 80–300) operated at 300 kV for soot classification. We calculated the morphological parameters with the freely available image processing software ImageJ^76^. For the image analysis, we used a Gaussian blur filter to smooth the edges of the binarized images. The results of these analyses are reported in section 4 of the SI.

A High Resolution Time of Flight Aerosol Mass Spectrometer (HR-ToF-AMS, Aerodyne Research Inc.)^77^ was used to measure the mass concentration of non-refractory components in submicron ambient aerosol (in μg m^−3^). Organic, sulphate, nitrate, ammonium, and chloride aerosol concentrations were measured with a resolution of 5 minutes in V- mode (mass resolution of about 2200 at m/z 28). Particles were dried with a Nafion drier before analysis (relative humidity below 30%). The collection efficiency was corrected based on aerosol chemical composition, according to Bahreini *et al*.^78^, and validated by comparison with particle size distribution data and sulfate off-line measurements. The mass concentration of black carbon equivalent (a surrogate for soot) in the ambient samples was measured with a 7-wavelength Aethalometer (Magee, AE31) with a time resolution of 5 minutes. Attenuation measurements were corrected according to Virkkula *et al*.^79^, and equivalent black carbon concentrations were calculated from attenuation data at 880 nm and assuming a mass absorption cross section equal to 16.6 m^2^ g^−1^, as suggested by the manufacturer.

To investigate soot aerosol sources, we analyzed aethalometer absorption coefficients with the Sandradewi *et al*.^80^ model. The model allowed us to quantify the contribution of traffic emission and wood burning emission to aerosol light absorption, assuming a constant and known absorption Ångström exponent for traffic and wood burning carbonaceous aerosol (0.90 and 1.68, respectively)^81^. The absorption coefficient assigned to traffic and wood burning by the model was on average 9.0 Mm^−1^ and 6.7 Mm^−1^ for the “sunny” sample period, and 6.0 Mm^−1^ and 3.9 Mm^−1^ for the “foggy” sample period, respectively. The traffic to wood burning ratios were quite similar (1.3 and 1.5), suggesting similar sources and aging of soot particles during the two cases studied.

### Ambient samples from other locations

In addition to the SPC, soot particles were collected at ten additional locations around the world. Sampling techniques used either aspiration or impaction (four-stage cascade impactors). Specimen collection media varied, but typically included polycarbonate membranes and formvar copper grids. Sample preparation and imaging conditions also varied. Information on sampling locations and conditions is provided in Table 1.

### Laboratory sample collection from the Pi Chamber

The name of the Pi Chamber derives from the interior volume of 3.14 m^3^ when a cylindrical insert is in place. A detailed discussion of the Pi Chamber is provided by Chang *et al*.^82^. In the Pi Chamber, clouds can be formed by expansion or by turbulent mixing. In the mixing mode, a long-lasting, steady-state cloud is formed by imposing a temperature gradient between the top and bottom surfaces (thermal plates), while maintaining the two surfaces saturated with respect to water. For the experiments discussed here, we generated a mixing cloud, using a temperature gradient of 17 K between the warmer bottom plate and the colder top plate to drive convection, a process that is described in detail elsewhere^83^. Soot particles were drawn from a kerosene flame using an eductor pump (AIR-VAC, model: AVR093M) driven by compressed clean and dry air, and injected into the Pi Chamber. We then formed a cloud by using the soot particles as cloud condensation nuclei. The LWC in the Pi Chamber during the experiment was ~0.085 g m^−3^ (measured using a phase Doppler interferometer, Dantec Dynamics), similar to that measured at SPC. We used a Pumped Counterflow Virtual Impactor (PCVI-8100, Brechtel Mfg.) to collect cloud droplets. In the PCVI, clean dry air pumped in the direction opposite (counterflow) to that of the input flow drives smaller interstitial aerosols away, allowing only larger particles (mainly droplets) to pass through the inlet, due to their inertia^84^. In other words, only particles with enough mass can overcome the counterflow, and pass through the sampling orifice. Because dry air is used in the counterflow, the droplets rapidly evaporate, leaving behind the residual soot particles. The PCVI was run in flow conditions to achieve a size cut of ~4.5 μm. To collect interstitial aerosol particles (*i*.*e*., soot which did not activate to become cloud droplets), we used a 6.4 mm outer diameter stainless steel tube which protrudes near to the center of the PI Chamber (~1 m). Both the residuals from the PCVI and the interstitial aerosol passed through a diffusion dryer to further remove moisture before being sampled or measured with a Scanning Mobility Particle Sizer (SMPS- TSI-3772). A schematic of the set-up is shown in Supplementary Fig. S11. In addition, during the same experiment, we collected a sample of the nascent soot particles (freshly emitted) before injecting them into the Pi Chamber to compare the morphology of nascent soot with that of interstitial soot. All samples were collected on 13 mm diameter nuclepore polycarbonate membranes, having a pore size of 0.1 μm (Whatman Inc., Chicago, Illinois, USA) using a custom-built sampler at a flow rate of 0.4 lpm. Single particles were imaged with a Hitachi S-4700 field emission scanning electron microscope at a magnification of 60–100 kX, an accelerating voltage of 1 kV, and a working distance of 4 mm.

Comparing the cloud droplet residual with the interstitial and nascent soot allows us to investigate and quantify the differences between the morphology of soot that took part in cloud processing compared with those that did not. We note, that both the interstitial and residual samples were exposed to RH values near100 %, therefore, the comparison with the nascent soot also allows to separately determine the effect of high RH within the timescale of the experiment.

### Uncertainties in the image processing

In addition to statistical errors, there are potential errors in the morphological parameters associated with image acquisition and image processing. Bhandari *et al*.^70^ estimated the errors in convexity and roundness to be 3.9% and 4.4%, respectively. Similarly, the errors in *L*_*max*_, *W*, and *d*_*p*_ were estimated to be 1.5%, 1.8%, and 14%, respectively. Using the errors in *L*_*max*_ and *W*, we calculated 1.9% uncertainty in the *AR*. The uncertainty in *D*_*Aeq*_ was estimated to be 3.2%. We calculated the total error by propagating all the errors (statistical error and errors associated with image acquisition and image processing) in quadrature.

## Supplementary information

Revised SI_soot Compaction
