# Supplementary material for: Extensive Soot Compaction by Cloud Processing from Laboratory and Field Observations
Source: Sci Rep. 2019 Aug 14;9:11824. doi: 10.1038/s41598-019-48143-y (PMC6694138; doi:10.1038/s41598-019-48143-y)
Supplement: Supplementary file 1 — Revised SI_soot Compaction [file 41598_2019_48143_MOESM1_ESM.pdf]

Supporting Information for

Extensive Soot Compaction by Cloud Processing from Laboratory and Field Observations

Janarjan Bhandari[1*], Swarup China[1,3], Kamal Kant Chandrakar[1], Greg Kinney[1], Will Cantrell[1], Raymond A. Shaw[1], Lynn R. Mazzoleni[2], Giulia Girotto[1], Noopur Sharma[1,3], Kyle Gorkowski[1,4,8], Stefania Gilardoni[5], Stefano Decesari[5], Maria Cristina Facchini[5], Nicola Zanca[5,14], Giulia Pavese[6], Francesco Esposito[7], Manvendra K. Dubey[8], Allison C. Aiken[8], Rajan K. Chakrabarty[9], Hans Moosmüller[10], Timothy B. Onasch[11], Rahul A. Zaveri[3], Barbara V. Scarnato[12], Paulo Fialho[13], Claudio Mazzoleni[1*]

[1] Atmospheric Sciences Program and Department of Physics, Michigan Technological University, Houghton, MI, USA
[2] Atmospheric Sciences Program and Department of Chemistry, Michigan Technological University, Houghton, MI, USA
[3] Pacific Northwest National Laboratory, Richland, WA, USA
[4] Atmospheric and Oceanic Sciences, McGill University, Montreal, Canada
[5] Institute of Atmospheric Sciences and Climate (CNR-ISAC), Italy
[6] Institute of Methodologies for Environmental analysis (CNR-IMAA), Italy
[7] School of Engineering – University of Basilicata, Italy
[8] Los Alamos National Laboratory, Los Alamos, NM, USA
[9] Department of Energy, Environmental and Chemical Engineering, Washington University in St. Louis, MO, USA
[10] Desert Research Institute, Reno, NV, USA
[11] Aerodyne Research Inc., Billerica, MA, USA
[12] DNV GL, Høvik, Norway
[13] Instituto de Investigação em Vulcanologia e Avaliação de Riscos - IVAR -University of Azores, Portugal
[14] Department of Chemistry and Institute for Atmospheric and Earth System Research (INAR), University of Helsinki, FI-00014, Finland

1. Aspect ratio, convexity, and roundness of soot particles

An example of a scanning electron microscope (SEM) image for a soot particle and a schematic representation of the aspect ratio *(AR)*, convexity, and roundness calculations from the respective binary image are shown in **Figures S1a**, **S1b**, and **S1c**, respectively.

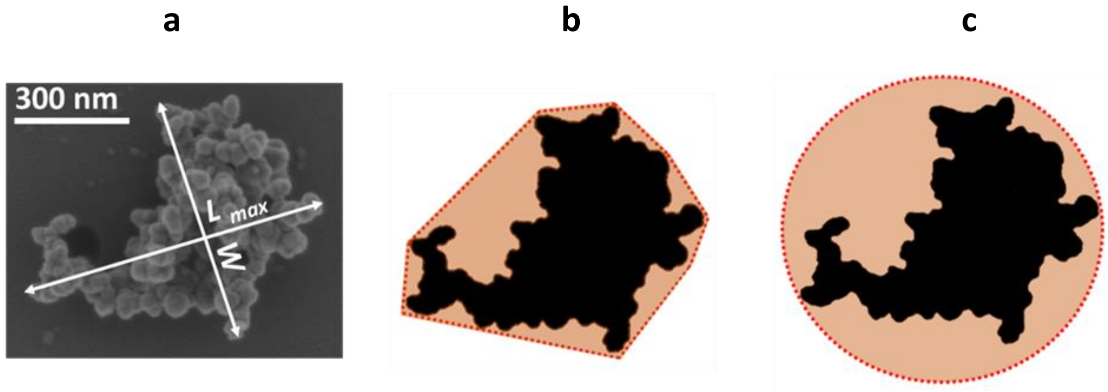

Aspect ratio =1.26 | Convexity = 0.67 | Roundness = 0.44

Figure S1. Example of soot particle collected in the laboratory. (**a**) SEM image and calculation of the aspect ratio from the maximum length L_{max} and the maximum width *W* of the particle (orthogonal to each other). (**b**) binarized image (in black) and schematic representation of the convexity calculation for the same soot particle shown in (a). The red dashed line represents the convex hull. (**c**) Schematic representation of the roundness calculation for the same soot particle shown in (a). The red dashed line represents the inscribing circle.

2. Meteorological conditions in San Pietro Capofiume (SPC)

Following are plots of temperature and relative humidity (**Figure S2**), and liquid water content and solar irradiance (**Figure S3**) during the sampling periods at the SPC site.

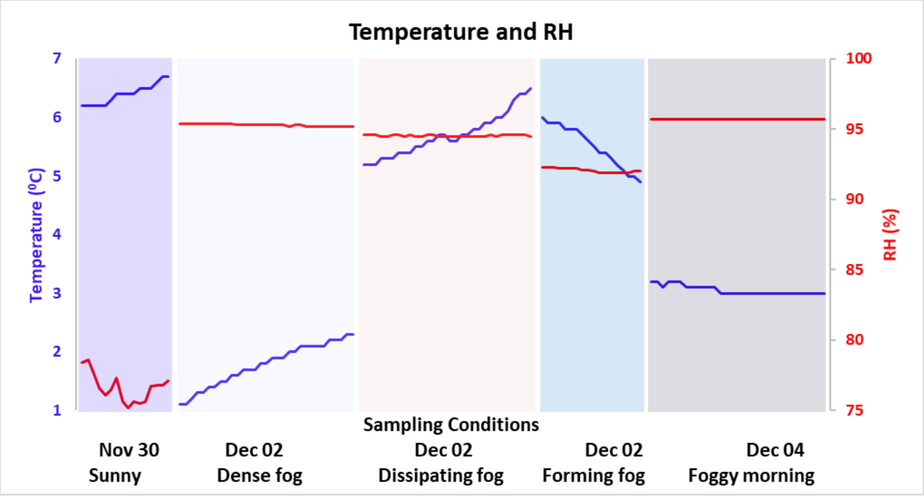

Figure S2. Temperature and relative humidity (RH) at the San Pietro Capofiume site in the Po Valley, during the collection of the five samples studied here.

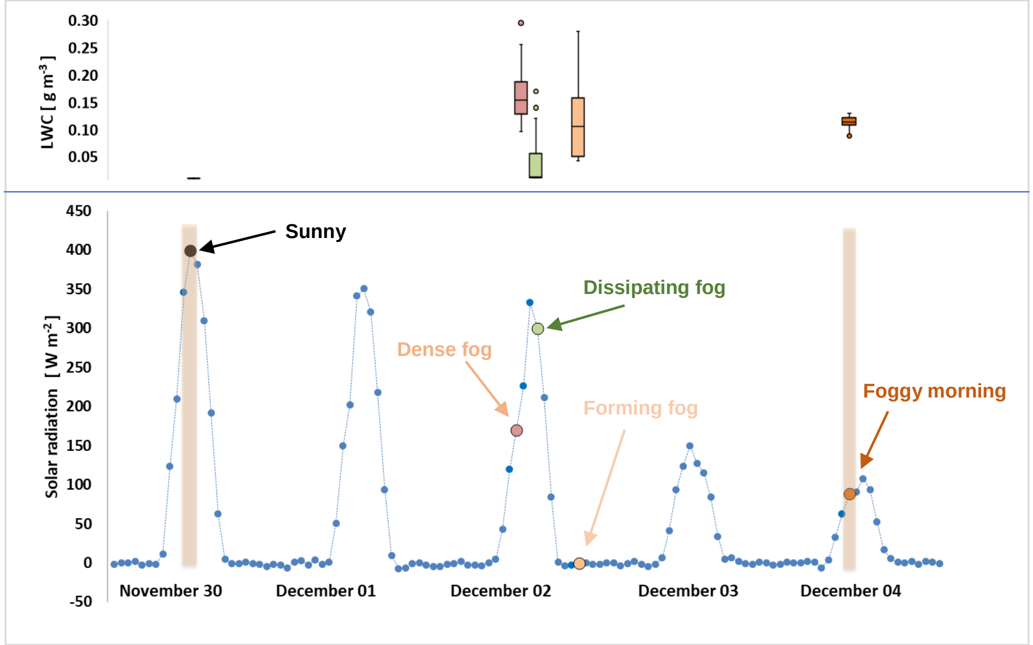

Figure S3. Liquid water content (LWC) (top) and solar irradiance (bottom) at the San Pietro Capofiume site during the period of collection of the five samples studied here. The boxplots for the LWC have the same color as the solar irradiance dots for the corresponding sampling periods. The upper and lower boundary of each box represent the third and first quartiles, respectively, and the horizontal bar in each box corresponds to the median. The whiskers represent the maximum and minimum values and the dots outside the whisker represent outliers. The shades represent the sampling periods for the two samples selected for the detailed study of the soot morphology. The solar irradiance data are hourly data points. Note that the boxplot corresponding to the sunny event appears as a horizontal bar due to nearly zero value of LWC (~0.01 g cm^{-3}) during the entire sampling period.

Table S1: Sampling conditions at the San Pietro Capofiume site. The first and the last samples in the table were those used for the detailed morphological analysis of soot.

**Event description**	**Sampling date and local time (2015)**	**Sampling flow rate [lpm]**	**Initial liquid water content [g m^{-3}]**	**Final liquid water content [g m^{-3}]**	**Average liquid water content [g m^{-3}]**
Sunny	30 November 10:40-10:55	0.13	0.01	0.01	0.01
Dense fog	2 December 9:40- 10:10	0.17	0.22	0.10	0.16
Dissipating fog	2 December 11:50-12:20	0.17	0.14	0.01	0.04
Forming fog	2 December 18:43-19:00	0.26	0.05	0.28	0.12
Dense fog in the morning (Foggy morning)	4 December 9:15-9:45	0.17	0.12	0.11	0.11

3. Mixing state of SPC soot particles

We visually classified the mixing state of more than 840 individual particles from SEM images of five SPC ambient samples. We also classified 351 soot particles from transmission electron microscopy (TEM) images of two additional samples collected during the sunny and the foggy morning events. We classified the soot particles based on the degree of coating. In brief, a bare or thinly coated soot particle, in which all the monomers are clearly visible, is classified as C0. In C1, a soot particle has some of the monomers covered by visibly thin coating, and in category C2, the soot particle has some monomers covered by thick coating; finally, in C3, most of the monomers are completely covered by the coating material and are barely distinguishable (**Figure S4a**). In some cases, the soot particles appear to be attached to, or partially engulfed by, other particles. Such partially encapsulated/surface attached soot is classified in an additional separate category, PE **(Figure S4b**). For example, the soot particle shown in **Figure S4b** is thickly coated (C2), and also attached onto the surface of another particle (PE) and is, therefore, categorized as PE-C2. In **Figure S4c** we show the classification of soot particles (from both SEM and TEM images) from different events (**Supplementary Table S1)**.

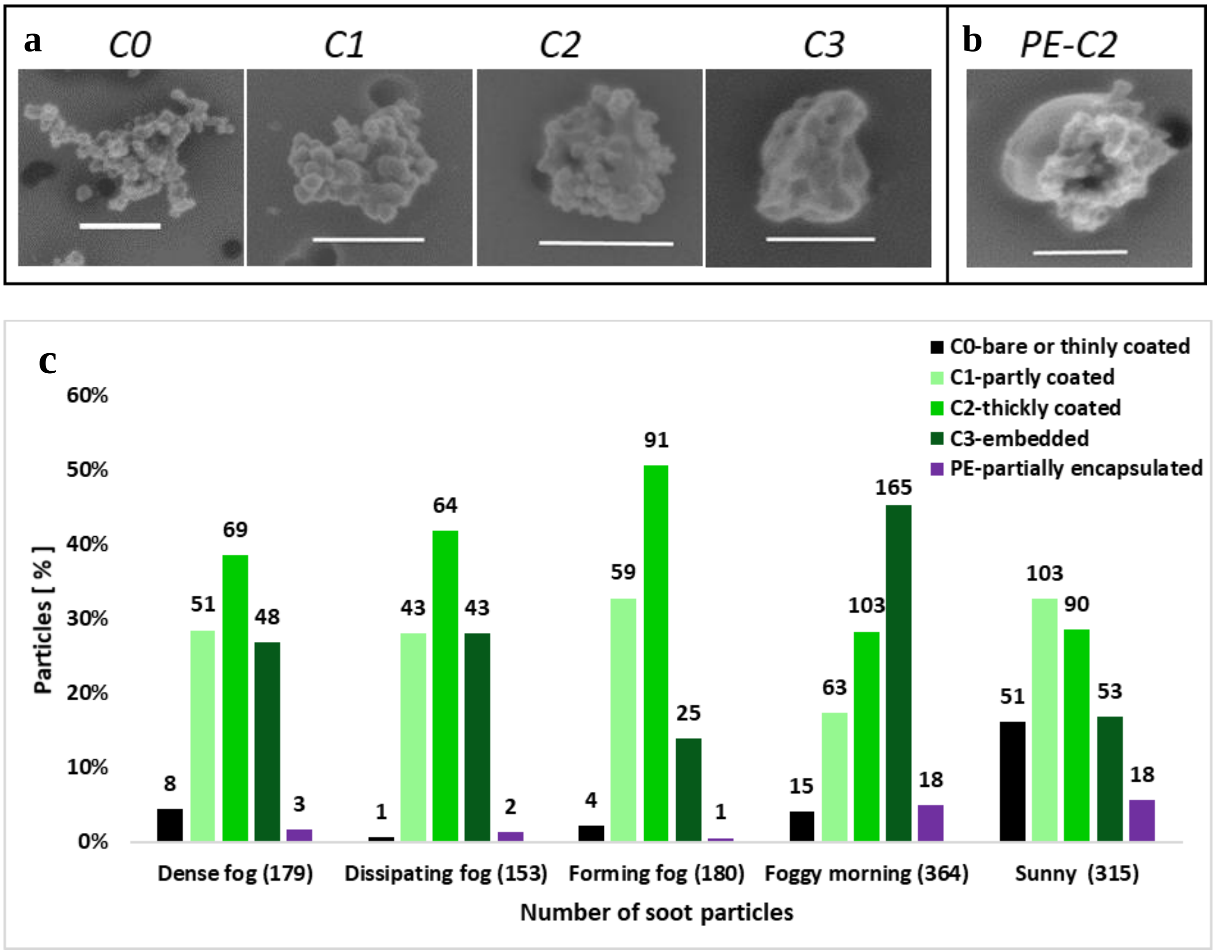

Figure S4. Mixing and classification of soot particles. (**a**) Soot particles with different degrees of coating classified to category C0 to C3. C0 – particle with clearly visible monomers, C1 – particle with some of the monomers covered by a visible amount of thin coating, C2 – particle with some of the monomers covered by thick coating,

and C3 – particle with most of the monomers covered by thick coating and barely distinguishable. (**b**) Partially encapsulated and thickly coated soot (PE-C2). The scale bar on each micrograph corresponds to 300 nm. (**c**) The contribution of soot particles for each category during different events. The total number of particles in each category is reported above each bar. In brakets is the total number of soot particles analyzed for each sample.

We note that this soot classification is based on a visual evaluation from the SEM micrographs, and therefore, it presents some subjectivity and some potential bias. For example, a heavily coated soot particle that would fall in category C3 might be excluded from the soot classification if none of the monomers in the SEM image were discernible. Also, the vacuum in the electron microscope chamber might result in evaporation of some of the most volatile coating material.

We found that most of the soot particles were partly or thickly coated (soot of category C1 or C2). A substantial fraction (15-45%) of soot in all samples were embedded (C3). These fractions suggest that soot was aged and mixed with other materials such as organics or sulfate, common in polluted regions [1,2]; finding consistent with the location of the sampling site located in a rural area at more than 10 km from major traffic roads. The aerosol mass spectrometer data showed ~30-45% organics, ~15-40% nitrate, and ~ 5% sulfate by mass during the collection periods of all five samples (**Figures S6** and **S7**). Electron energy Dispersive X-ray spectroscopy (EDX) analysis of individual soot particles from the sunny and foggy morning events showed an appreciable abundance of sulfur in both samples (**Figure S8**). The average (± standard deviation) S/C values were 0.010 (0.006) and 0.009 (0.005) for the sunny and foggy morning events, respectively. The presence of sulfur in the soot suggests that these particles were likely able to participate in cloud activation [3,4]. In all events, the number fraction of soot in category PE resulting from coagulation [5], was small (< 5%).

4. Single particle morphological analysis for soot collected at the SPC site

In **Table S2** we summarize the morphological results of the single particle analysis on the soot particles collected during the sunny and the foggy morning events at the SPC site.

**Parameter**	**Sunny: 109 particles**<br>**Mean ± S.D. (S.E.) [total error]**	**Foggy morning: 144 particles**<br>**Mean ± S.D. (S.E.) [total error]**
Convexity	0.71 ± 0.13 (0.01) [0.03]	0.80 ± 0.11 (0.01) [0.03]
Roundness	0.43 ± 0.15 (0.01) [0.02]	0.52 ± 0.14 (0.01) [0.03]
L_{max} **(nm)**	529 ± 290 (28) [29]	343 ± 150 (13) [14]
W **(nm)**	330 ± 200 (19) [20]	227 ± 95 (7.9) [8.9]
AR	1.66 ± 0.45 (0.04) [0.05]	1.53 ± 0.34 (0.03) [0.04]
d_m **(nm)**	38.7 ± 10.2 (1.4) [5.6]	35.7 ± 6.7 (0.56) [5.0]
D_{Aeq} **(nm)**	326 ± 155 (15) [18]	237 ± 93 (8) [11]

Table S2. Morphological parameters for particles from the sunny and the foggy morning events for soot in category C0 and C1 only, with mean, standard deviation (S.D.), standard error (S.E.), and total error values. The total error (in square brackets) is calculated by adding all the errors in quadrature, as discussed in the method section.

Since fresh soot particles have a fractal-like structure [6], the mass of soot scales with its length following a power law as given by Mandelbrot and Pignoni [7]. Because the mass of soot is proportional to the number of monomers N in the aggregate, this scaling law is often expressed as:

$$N = k_g \left(\frac{2R_g}{d_m}\right)^{D_f} \qquad \text{(SI-1)}$$

where K_g is the fractal prefactor, R_g is the radius of gyration of the aggregate, and d_m is the arithmetic mean diameter of the monomers. The exponent D_f is the fractal dimension. Lacy soot aggregates have lower values of D_f than compact soot particles. The ensemble method is commonly used to calculate D_f for lacy aggregates when $D_f < 2$. In the ensemble method N values for several soot particles are plotted as a function of their radius of gyration (or a surrogate of it) and a power law is fitted to the data [6,8,9]. However, for the purpose of our study, we are also interested in highly compact soot particles with $D_f > 2$. For such compact soot aggregates, the use of the ensemble method would provide erroneous results due to an underestimation of N calculated from 2-D (projected) images [10]. Thus, we calculated what we call a 2-D ensemble fractal dimension (D_{2f}) (**Figure S5**) where the particle projected area (A_p) scales with one of its length scales, here chosen to be L_{max}, similarly to the scaling law given by Lee and Kramer [11]:

$$A_p = K_{2g} \, (L_{max})^{D_{2f}} \qquad \text{(SI-2)}$$

where K_{2g} is a "two-dimensional" prefactor and L_{max} is the maximum length of the aggregate.

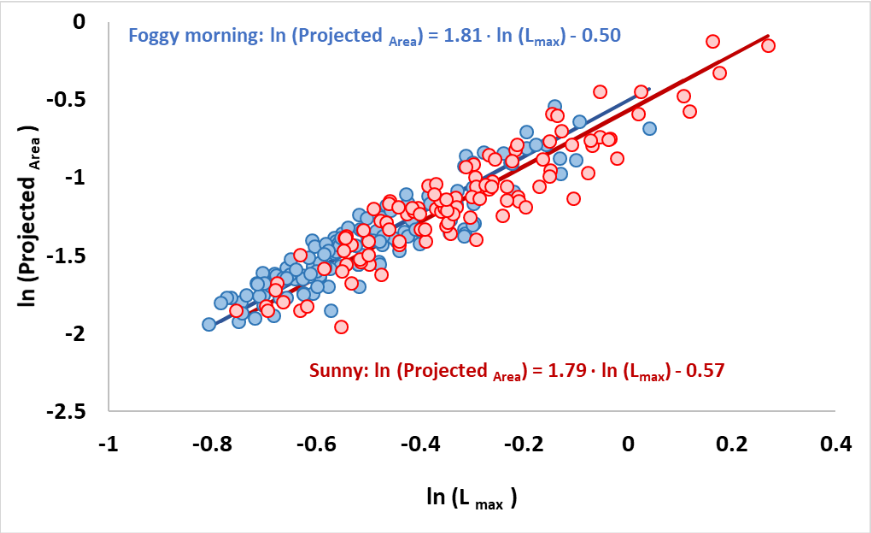

Figure S5. Scaling law fits for sunny and foggy morning events. Lines represent linear fits in log-log space using a modified orthogonal regression [12], the slope represents the D_{2f}.

The D_{2f} values for the samples from the sunny and the foggy morning events are listed in **Table S3**.

**Sample**	**D_{2f} with S.D.**	**Number of particles**
**Sunny event**	1.79 (0.01)	109
**Foggy morning event**	1.81 (0.02)	144

Table S3. 2-D fractal dimension and standard deviation (S.D.) of soot particles collected at SPC during the sunny and the foggy morning events.

It should be noted that, although the D_{2f} for the sunny event is indeed smaller than the D_{2f} for foggy events as expected, the difference is very small and not statistically significant. This reflects the fact that different sources and soot at different aging stages contributed to the ambient samples, making the ensemble approach needed for the D_{2f} estimate less appropriate and sensitive for detecting changes in morphology than non-ensemble parameters such as the convexity and roundness.

Figures S6 and S7. Mass fractions and concentrations of different aerosol components measured during the sampling periods at SPC with an aerosol mass spectrometer and an aethalometer.

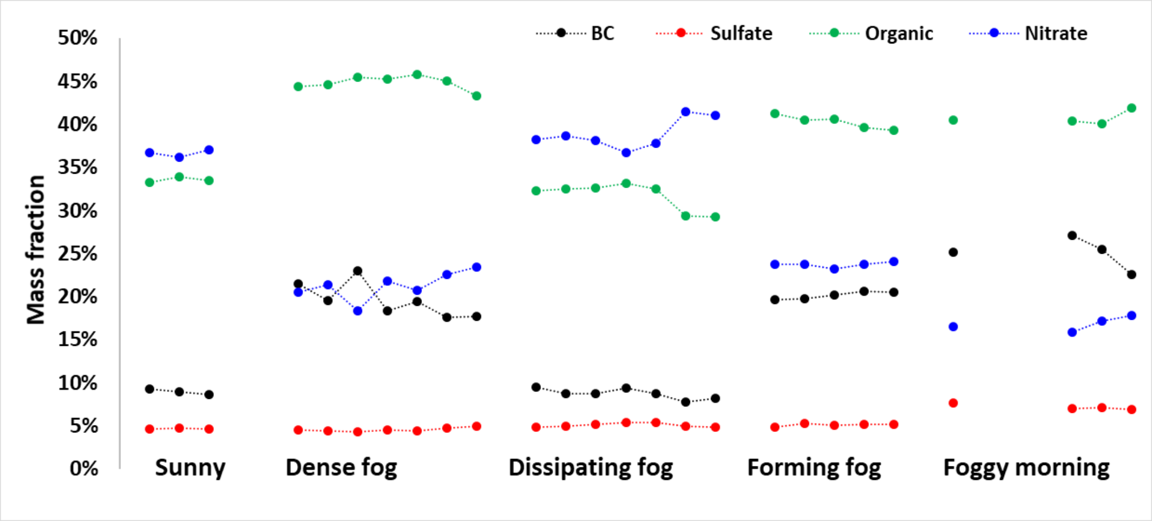

Figure S6. Aerosol relative composition and black carbon fraction for the sunny and foggy events. Data were taken at 5-minutes intervals.

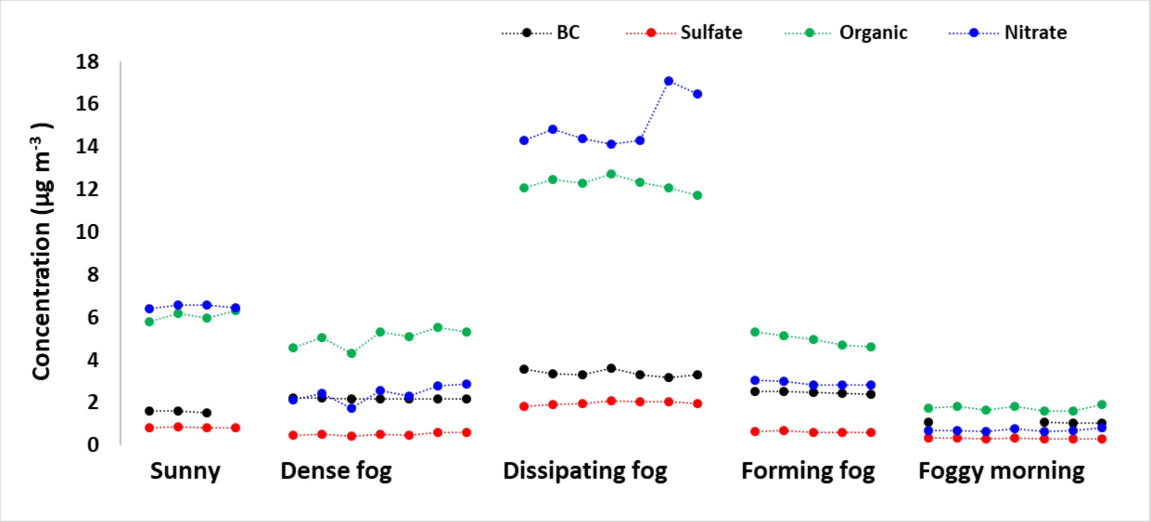

Figure S7. Aerosol composition and black carbon mass concentrations for the sunny and foggy events. Data were taken at 5-minutes intervals.

Atomic number percent ratios of sulfur and carbon in ambient soot samples collected at SPC during the sunny and foggy morning events, determined with EDX spectroscopy, are shown in **Figure S8**.

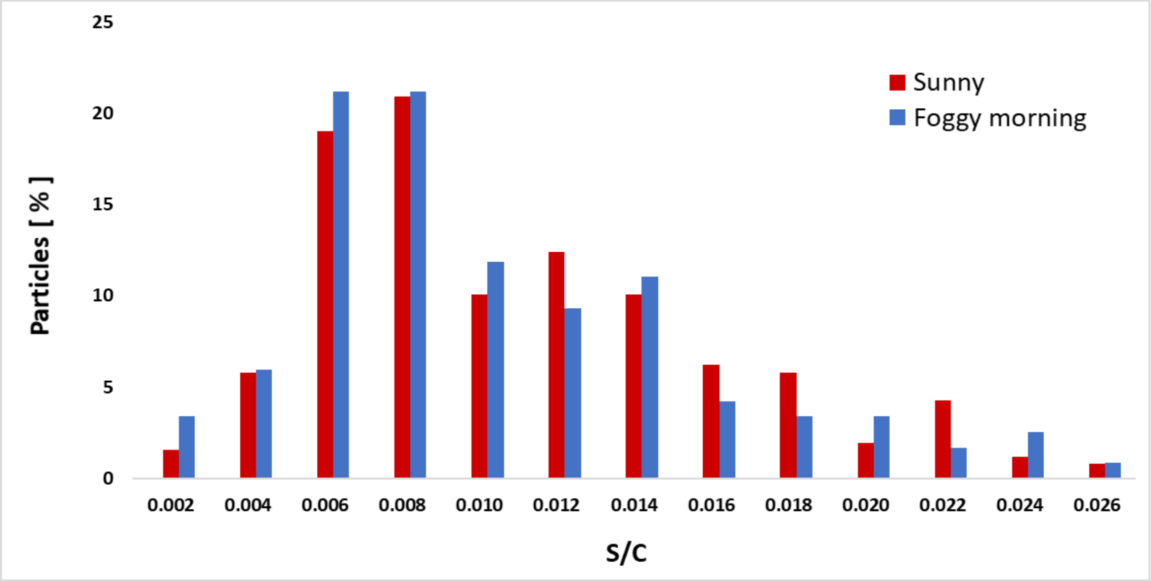

Figure S8. Sulfur to carbon atomic number percent ratios in coated soot for SPC samples from the foggy morning and the sunny events.

Distribution of aspect ratio and area equivalent diameter for of soot particles from the sunny and foggy morning events at SPC are shown in **Figures S9** and **S10**.

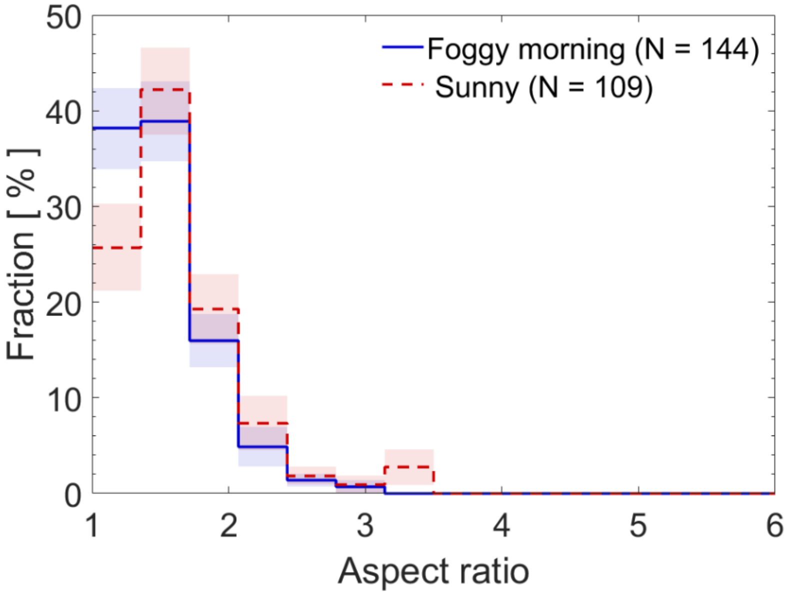

Figure S9. Distribution of aspect ratio of soot particles in category C0 and C1 for sunny and foggy morning events. The colored bands represent 68% confidence intervals. The total number fraction of particles for each distribution is normalized to 100%.

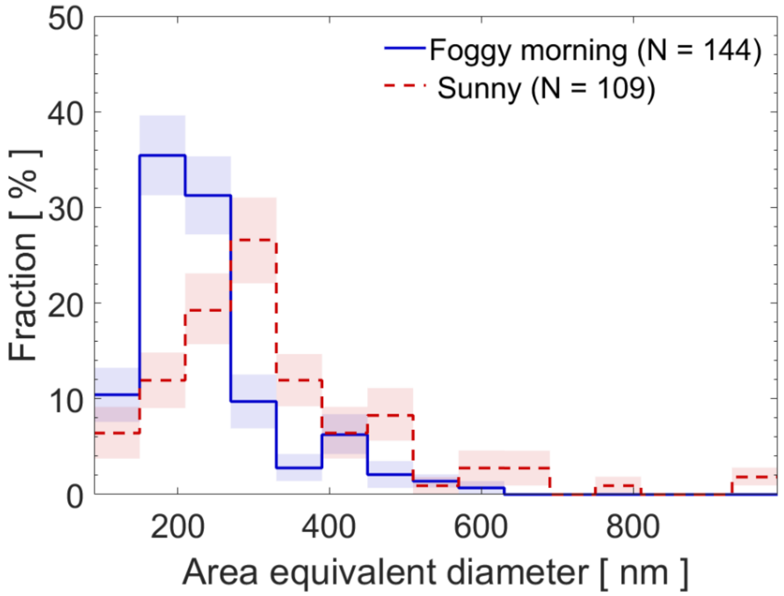

Figure S10. Distribution of the area equivalent diameter of soot particles of category C0 and C1 for sunny and foggy morning events. The colored bands represent 68% confidence intervals. The total number fraction of particles for each distribution is normalized to 100%.

5. **Samples collection and morphological parameters of soot from the Pi Chamber experiments**

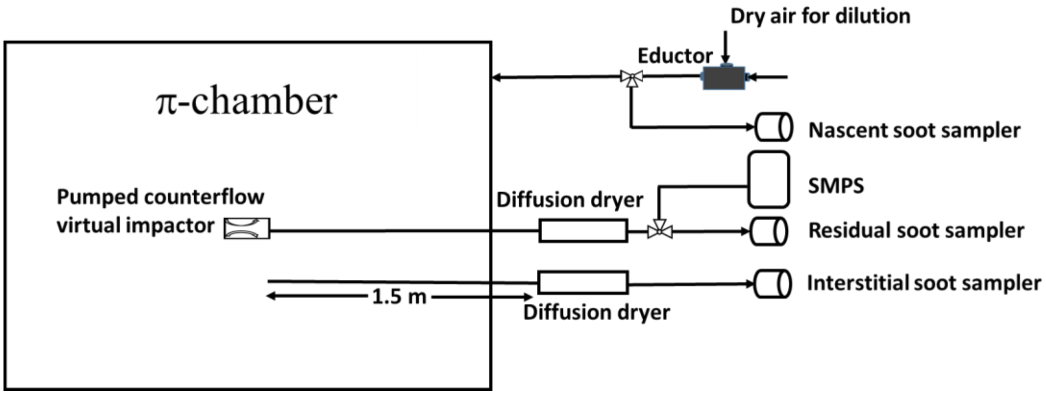

Figure S11. Soot aerosol sampling set-up in the Pi Chamber.

Nascent, interstitial, and residual soot samples were collected from the Pi Chamber. By 'nascent', we refer to the kerosene flame generated fresh soot particles that were collected directly from the source after dilution with dry air. In **Table S4** we report the soot morphological parameters for the samples collected during the laboratory experiments carried out in the Michigan Tech Pi-Chamber for: 1) nascent soot, 2) interstitial soot, 3) residual soot.

**Parameter**	**Nascent soot: 126 particles Mean ± S.D. (S.E.) [total error]**	**Interstitial soot: 161 particles Mean ± S.D. (S.E.) [total error]**	**Residual soot: 160 particles Mean ± S.D. (S.E.) [total error]**
Convexity	0.59 ± 0.12 (0.01) [0.03]	0.56 ± 0.13 (0.01) [0.02]	0.78 ± 0.14 (0.01) [0.03]
Roundness	0.30 ± 0.12 (0.01) [0.02]	0.29 ± 0.12 (0.01) [0.02]	0.48 ± 0.16 (0.01) [0.02]
L_{max} (nm)	675 ± 468 (42) [43]	491 ± 240 (19) [20]	300 ± 190 (15) [16]
W (nm)	331 ± 155 (14) [15]	260 ± 130 (10) [11]	186 ±110 (8.4) [9.1]
AR	2.02 ± 0.67 (0.06) [0.07]	1.95 ± 0.6 (0.05) [0.06]	1.63 ± 0.44 (0.03) [0.05]
d_m (nm)	32.9 ± 5.6 (0.70) [4.7]	27.1 ± 4.9 (0.52) [3.8]	29.2 ± 7.2 (1.0) [4.2]
D_{Aeq} (nm)	328 ± 135 (12) [16]	239 ± 89 (7) [10]	192 ± 96 (8) [10]

Table S4. Morphological parameters for nascent, interstitial, and residual soot particles with mean, standard deviation (S.D.), standard error (S.E.), and total error values. The total error (in square brackets) is calculated by adding all the errors in quadrature, as discussed in the method section.

We also calculated the D_{2f} for nascent, interstitial and cloud residual soot particles to quantify soot restructuring and compaction by cloud processing. The fits are shown in **Figures S12a** and **S12b** where the logarithm of A_p is plotted as a function of the logarithm of L_{max}. The slope for the residual soot particles (D_{2f}=1.63) is higher than for the interstitial particles (D_{2f} =1.54) and nascent particles (D_{2f} =1.50) indicating compaction of soot particles after cloud processing. In earlier studies, higher values of D_f have also been reported for soot particles after cloud processing e.g., [13,14].

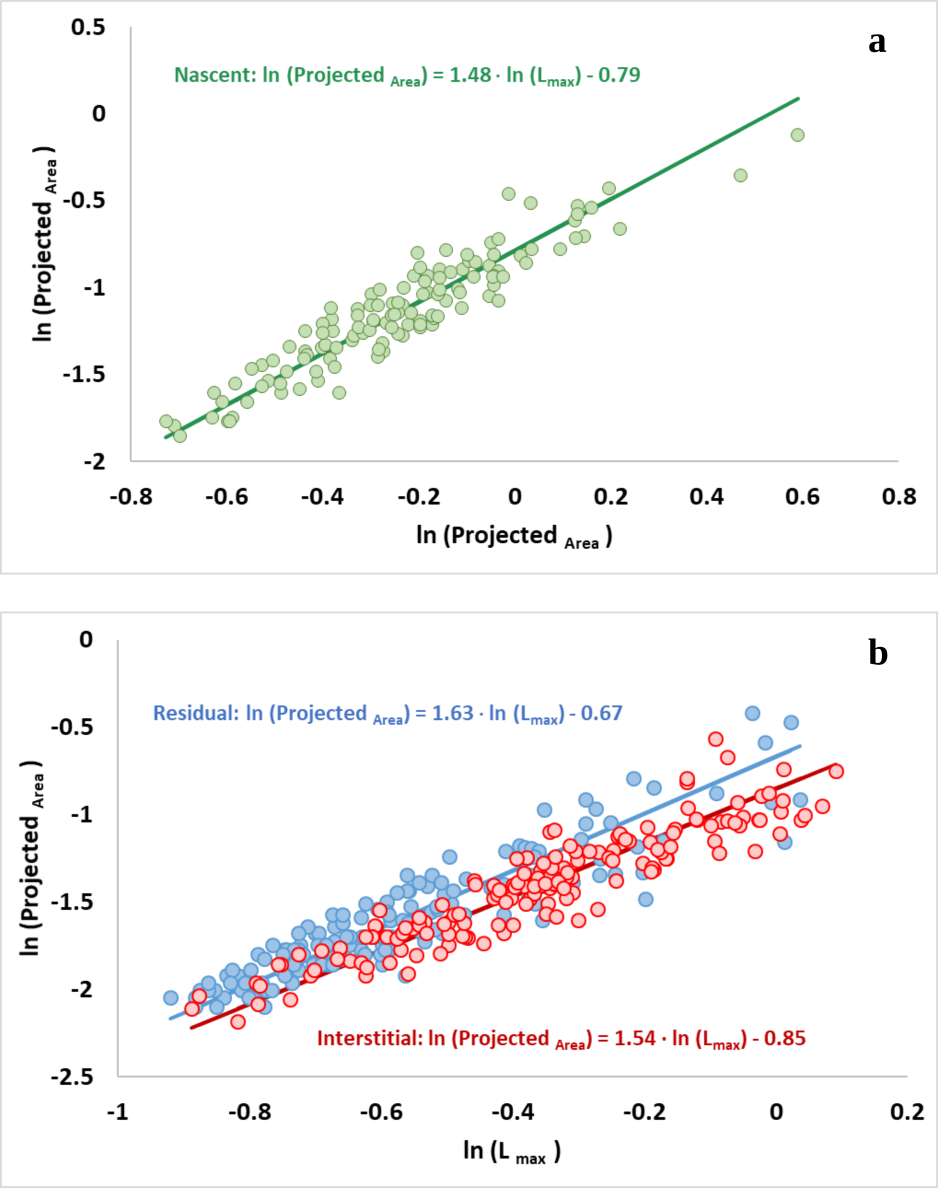

Figure S12. Scaling law fits for (**a**) nascent, (**b**) interstitial, and residual soot particles. The lines represent the linear fits in log-log space using a modified orthogonal regression [12], the slope represents the D_{2f}. D_{2f} for the interstitial and residual are shown together to compare to each other.

The D_{2f} values for nascent, interstitial and residual soot particles are summarized in **Table S5**.

**Sample**	**D_{2f} with S.D.**	**Number of particles**
**Nascent**	1.50 (0.02)	126
**Interstitial**	1.54 (0.02)	161
**Residual**	1.63 (0.01)	160

Table S5. 2-D fractal dimension and standard deviation for soot particles collected during the Pi Chamber experiments.

Distribution plots for the aspect ratio and area equivalent diameter of interstitial and residual soot particles collected from the Pi Chamber are shown in **Figures S13** and **S14**.

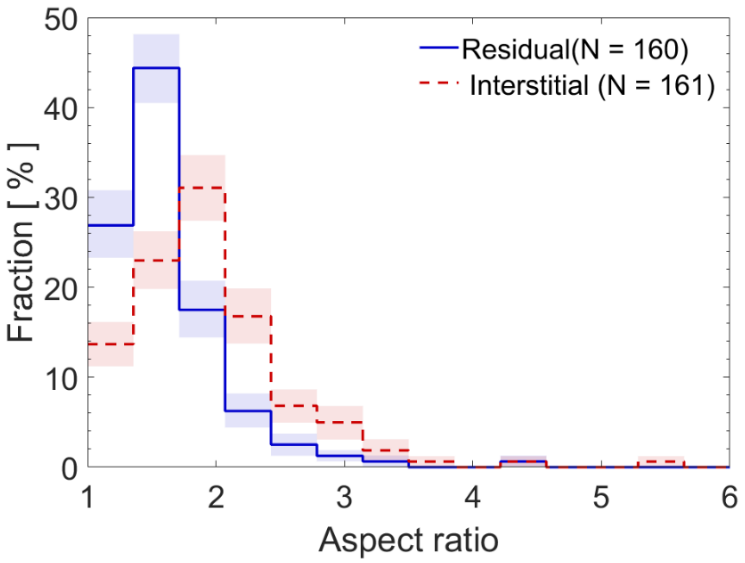

Figure S13. Distribution of aspect ratio of soot particles for interstitial and residual soot samples from the Pi Chamber. The colored bands represent 68% confidence intervals. The total number fraction of particles for each distribution is normalized to 100%.

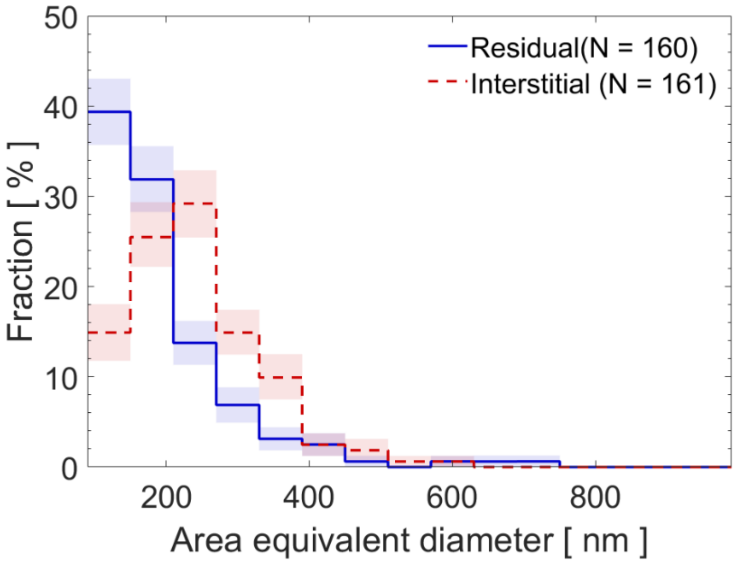

Figure S14. Distribution of the area equivalent diameter of soot particles for interstitial and residual samples from the Pi Chamber. The colored bands represent 68% confidence intervals. The total number fraction of particles for each distribution is normalized to 100%.

Finally, we compare the roundness and convexity of nascent kerosene soot particles with the interstitial particles collected during the Pi Chamber experiments. Convexity and roundness showed similar distributions for nascent and interstitial particles as shown in **Figures S15** and **S16**.

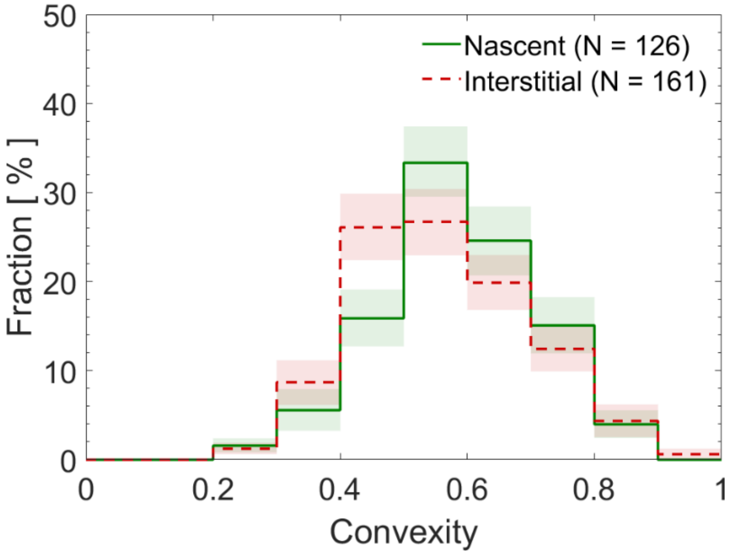

Figure S15. Distributions of the convexity for nascent and interstitial soot particles from the Pi Chamber. The colored bands represent 68% confidence intervals. The total number fraction of particles for each distribution is normalized to 100%.

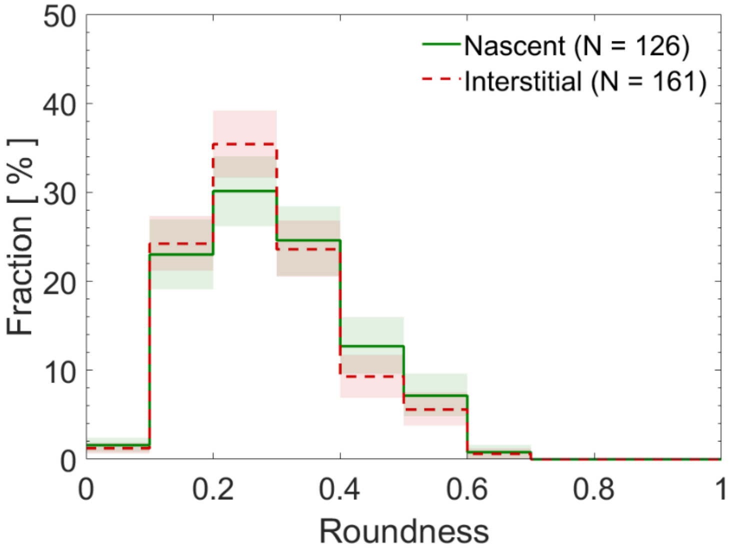

Figure S16. Distributions for the roundness of nascent and interstitial soot particles from Pi Chamber. The colored bands represent 68% confidence intervals. The total number fraction of particles for each distribution is normalized to 100%.

6. Roundness of soot particles at several locations

In **Figure S17**, we report the distributions of roundness in terms of histograms and box plots, at different locations around the world, similar to the convexity distributions map shown in **Figure 5** in the main paper.

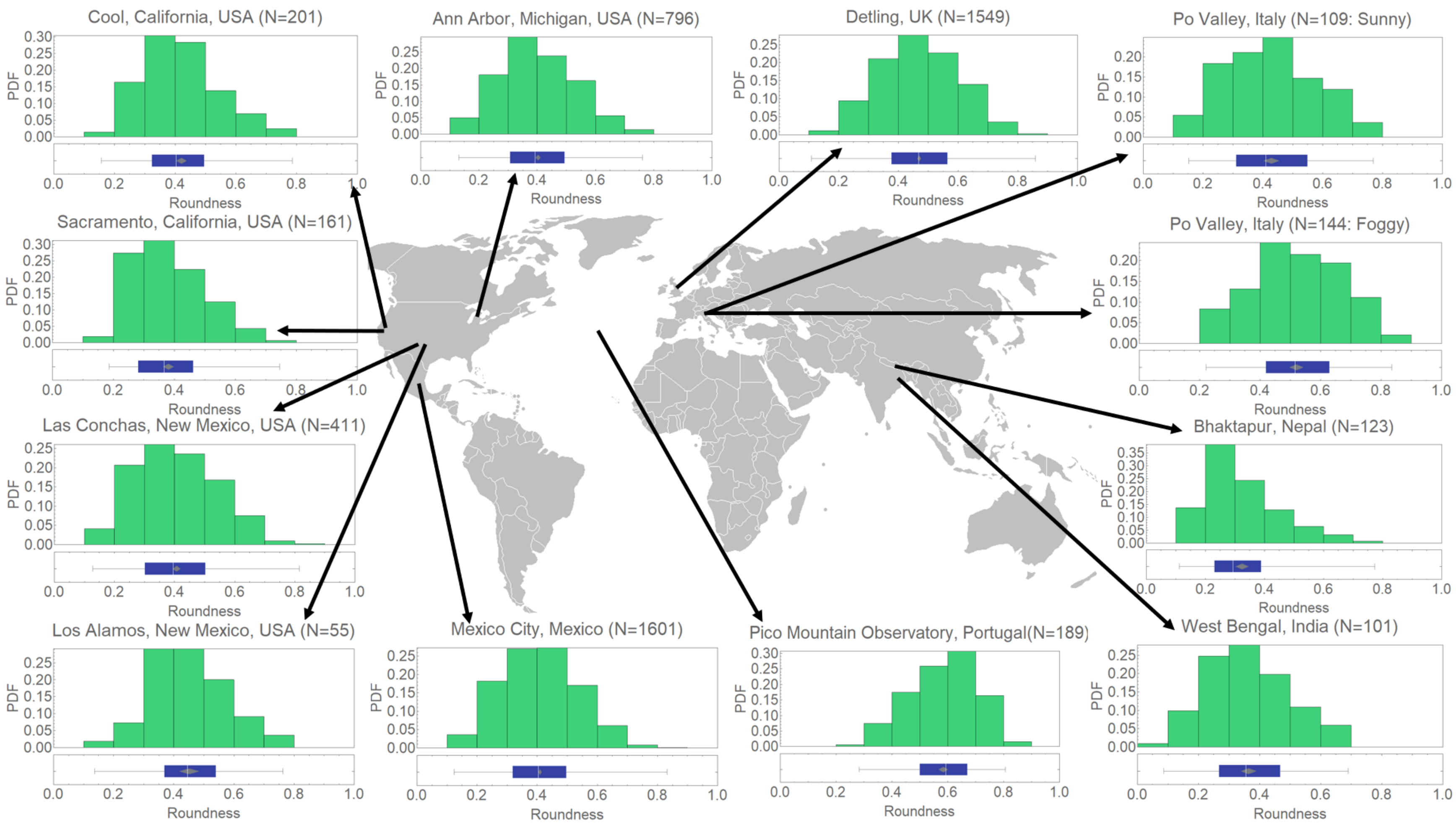

Figure S17. Roundness histograms and box plots for thinly coated soot particles from different locations. On each box plot, the vertical white line represents the median and the grey diamond represents the mean confidence interval for each distribution, the box extremities represent 25% and 75% quantiles and the whiskers represent the lower and upper values. For each distribution, N (in brackets) is the number of soot particles analyzed, and PDF on the y-axis is the probability distribution of particles for each roundness bin.

7. Convexity and roundness of soot particles with aging

Finally, in **Figures S18** and **S19**, we report the mean convexity and roundness values for soot particles for increasing estimated aging time. The circles indicate ambient samples, the triangles (liquid water) and asterisks (ice) represent laboratory cloud processing experimental results. The blue and green color indicate urban and biomass dominating sources, respectively, while the red color indicates samples affected by cloud processing. The arrow is just to suggest a potential trend with age, but it is evident that cloud processing can cause significant compaction even at very short aging time.

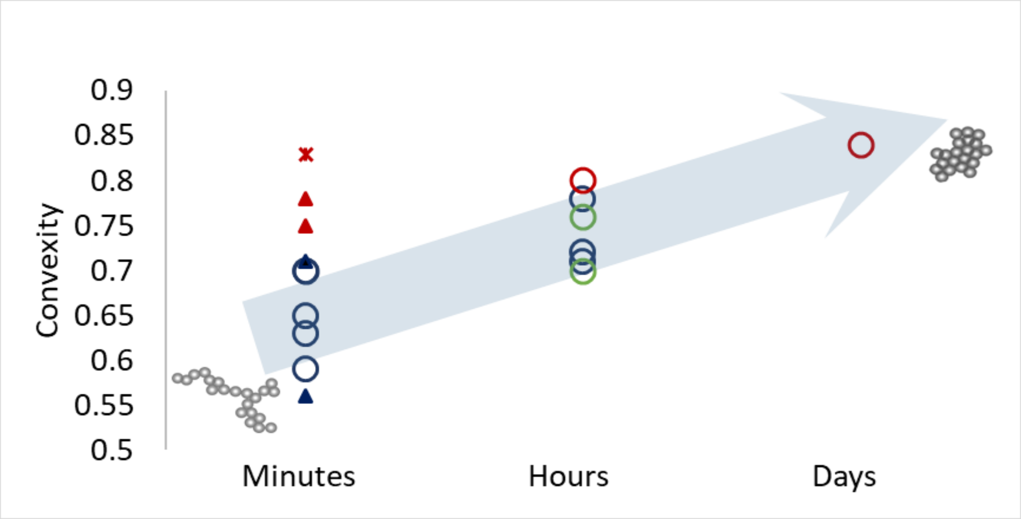

Figure S18. Convexity of ambient soot samples (circles) and laboratory studies (triangles and asterisk) from **Table 2** in the main text. The red asterisk specifically indicates ice processing. Blue circles indicate urban-influenced samples, red circles indicate samples affected by cloud processing, green circles indicate samples from biomass burning plumes. The x-axis is only a semi-quantitative estimate of the mean age of the sampled particles. The arrow is just suggestive of a trend.

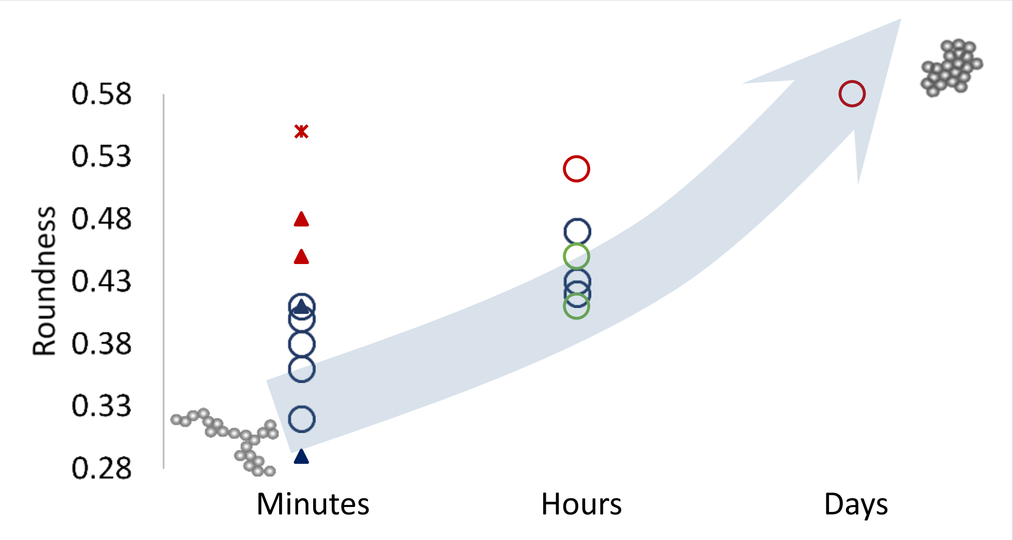

Figure S19. Same as **Figure S18** but for roundness.

References

1 Adachi, K., Chung, S. H. & Buseck, P. R. Shapes of soot aerosol particles and implications for their effects on climate. *Journal of Geophysical Research: Atmospheres* **115** (2010).

2 Peng, J. *et al.* Markedly enhanced absorption and direct radiative forcing of black carbon under polluted urban environments. *Proceedings of the National Academy of Sciences* **113**, 4266-4271 (2016).

3 Zhang, G. *et al.* The single-particle mixing state and cloud scavenging of black carbon: a case study at a high-altitude mountain site in southern China. *Atmospheric Chemistry and Physics* **17**, 14975-14985 (2017).

4 Zhang, R. *et al.* Variability in morphology, hygroscopicity, and optical properties of soot aerosols during atmospheric processing. *Proceedings of the National Academy of Sciences* **105**, 10291-10296 (2008).

5 Sharma, N. *et al.* Physical Properties of Aerosol Internally Mixed With Soot Particles in a Biogenically Dominated Environment in California. *Geophysical Research Letters* **45**, (2018).

6 Oh, C. & Sorensen, C. The effect of overlap between monomers on the determination of fractal cluster morphology. *Journal of Colloid and Interface Science* **193**, 17-25 (1997).

7 Mandelbrot, B. B. & Pignoni, R. *The Fractal Geometry of Nature*. Vol. 173 (WH freeman New York, 1983).

8 Brasil, A., Farias, T. L. & Carvalho, M. A recipe for image characterization of fractal-like aggregates. *Journal of Aerosol Science* **30**, 1379-1389 (1999).

9 China, S., Salvadori, N. & Mazzoleni, C. Effect of traffic and driving characteristics on morphology of atmospheric soot particles at freeway on-ramps. *Environmental Science & Technology* **48**, 3128-3135 (2014).

10 China, S. *et al.* Morphology and Mixing State of Aged Soot Particles at a Remote Marine Free Troposphere Site: Implications for Optical Properties. *Geophysical Research Letters* **42**, 1243-1250 (2015).

11 Lee, C. & Kramer, T. A. Prediction of three-dimensional fractal dimensions using the two-dimensional properties of fractal aggregates. *Advances in Colloid and Interface Science* **112**, 49-57 (2004).

12 Mazzoleni, C. *et al.* On-Road Vehicle Particulate Matter and Gaseous Emission Distributions in Las Vegas, Nevada, Compared with Other Areas. *Journal of the*

Air & Waste Management Association **54**, 711-726, doi:10.1080/10473289.2004.10470938 (2004).

13 Colbeck, I., Appleby, L., Hardman, E. & Harrison, R. M. The optical properties and morphology of cloud-processed carbonaceous smoke. *Journal of Aerosol Science* **21**, 527-538 (1990).

14 Zuberi, B. *et al.* Hydrophilic properties of aged soot. *Geophysical Research Letters* **32** (2005).
